# Electroplated Al Press Marking for Wafer-Level Bonding

**DOI:** 10.3390/mi13081221

**Published:** 2022-07-30

**Authors:** Muhammad Salman Al Farisi, Takashiro Tsukamoto, Shuji Tanaka

**Affiliations:** 1 Department of Robotics, Graduate School of Engineering, Tohoku University, Sendai 980-8579, Japan; takashiro.tsukamoto@tohoku.ac.jp (T.T.); mems@tohoku.ac.jp (S.T.); 2 Department of Biomedical Information Sciences, Hiroshima City University, Hiroshima 731-3194, Japan; 3 Micro System Integration Center (µSIC), Tohoku University, Sendai 980-8579, Japan

**Keywords:** wafer bonding, aluminum, electroplating, press marking, MEMS

## Abstract

Heterogeneous integration of micro-electro mechanical systems (MEMS) and complementary metal oxide semiconductor (CMOS) integrated circuits (ICs) by 3D stacking or wafer bonding is an emerging approach to advance the functionality of microdevices. Aluminum (Al) has been of interest as one of the wafer bonding materials due to its low cost and compatibility with CMOS processes. However, Al wafer bonding typically requires a high temperature of 450 ${}^{{}^{\circ}}$C or more due to the stable native oxide which presents on the Al surface. In this study, a wafer bonding technique for heterogeneous integration using electroplated Al bonding frame is demonstrated. The bonding mechanism relies on the mechanical deformation of the electroplated Al bonding frame through a localized bonding pressure by the groove structures on the counter wafer, i.e., press marking. The native oxide on the surface was removed and a fresh Al surface at the bonding interface was released through such a large mechanical deformation. The wafer bonding was demonstrated at the bonding temperatures of 250–450 ${}^{{}^{\circ}}$C. The influence of the bonding temperature to the quality of the bonded substrates was investigated. The bonding shear strength of 8–100 MPa was obtained, which is comparable with the other Al bonding techniques requiring high bonding temperature.

## 1. Introduction

Micro-electro mechanical systems (MEMS) is a class of transducers which is able to sense or manipulate physical, optical, or chemical quantities. To bridge the physical and cyber world, MEMS transducers are often coupled with complementary metal oxide semiconductor (CMOS) integrated circuits (ICs) which enables the electrical signal transmission with an adequate intelligence [1]. Most of the signal processing functions, such as amplification, temperature compensation, filtering, analog-to-digital conversion, storage, and communication are often performed by ICs in the integrated microsystems.

The advancement of the smart society driven by the internet of things (IoT) has enhanced the development of microfabricated devices, in particular for ubiquitous sensing to gather as much information as possible from the environment. Sensors, actuators, and ICs are demanded in a higher volume than ever. To date, the size of these devices have been scaled down in accordance to the Moore’s law. Such a miniaturization scheme has reduced the production cost of each chip. However, the miniaturization scheme is currently approaching its limit in the technological and economical aspects [2,3].

Heterogeneous integration by 3D stacking of microdevices is a promising alternative route when the device miniaturization according to the Moore’s law is approaching its limit [4]. Wafer bonding enables not only the integration of multiple devices in a small volume, but also device packaging at the same time [5]. In addition, each device can be fabricated separately at the wafer-level within the batch process before integrated at the final step, which maximizes the degree of freedom in the fabrication technique of each device.

The bonding material and the bonding technique are among the important aspects of the wafer bonding. Metallic materials are often of interest as a wafer bonding medium instead of their organic or glass counterparts due to their inherent capability as electrical interconnects simultaneously and high reliability [6,7,8]. Metal-based bonding techniques can be generally classified into transient liquid phase (TLP) or solid–liquid interdiffusion (SLID) and solid-state bondings. In the TLP bonding, an intermetallic joint is formed by a metallurgical reaction between a low melting point metal interlayer and a high melting point metal [9]. Such a reaction involving multiple metals helped to achieve a a low bonding temperature, which is useful to limit the thermo-mechanical stress related failure in the device. However, the metallurgical reaction often generates voids in the bonding interface, which may degrade the reliability of the package.

Solid-state metal bonding based on the same metal is another class of metal bonding techniques. General characteristics of conventional solid-state metal bonding technologies are summarized in Table 1. Room temperature direct bonding technologies are of interest due to their low bonding temperature. One approach is by performing the bonding process consecutively in the same chamber without breaking the vacuum after the deposition of the bonding layer [10]. Such a consecutive deposition and bonding process allowed the diffusion of high purity metals without any contamination, and, therefore, enabled the wafer bonding even without temperature elevation. Another approach is by utilizing a sub-nm very thin nanocrystalline metallic film [11]. Taking advantage of the higher diffusivity through the grain boundary, the nanocrystalline films of various metallic materials diffused at even a room temperature. However, in both techniques, the metallic material is deposited all over the wafer without allowing patterning steps or any intermediary processes before the bonding, which limits the practical applications.

As a wafer bonding material, Au is widely used owing to its high stability against process condition [12,13]. Furthermore, Au does not easily form a native oxide layer upon exposure to atmospheric pressure. Such qualities made Au available for thermocompression bonding without any need of surface pre-treatment prior to the bonding process. However, Au is often an unwanted material in CMOS process due to its high diffusivity. It often contaminates the other components, and, thus, the Au process lines are often separated from the main CMOS line. Such a requirement hindered the widespread usage of Au in CMOS process. In addition, Au is rather an expensive material.

Cu is often used as an alternative material [14]. It has a lower stability against various chemicals and processes in comparison to Au. For instance, some commercial photoresist strippers can unintentionally attack the Cu film. However, it has a significantly cheaper cost in comparison to Au. Meanwhile, Cu readily forms a native oxide upon exposure to the atmospheric environment. Its native oxide often hinders the bonding process. To remove the native oxide, various treatments, such as wet acid dipping, vapor, and plasma treatments, have been proposed to be performed prior to the bonding process [15]. However, Cu also often contaminates other components, and, thus, a barrier layer is often required to accompany its utilization.

Al is a promising alternative as a CMOS-friendly material for the wafer bonding process [16]. However, Al readily forms a native oxide upon exposure to the atmospheric environment. The aluminum oxide is one of the well-known stable metal oxide forms, which requires a high energy to be broken. A high bonding temperature of 450 ${}^{{}^{\circ}}$C or higher is generally required to break the Al native oxide and form a reliable Al bonding [17]. However, such a high bonding temperature may cause a device failure due to thermal-related effects and the coefficient of thermal expansion mismatch between the materials in the device.

To reduce the bonding temperature, several efforts have been made. For instance, by applying a high bonding pressure or prolonging the bonding duration, bonding at around 300 ${}^{{}^{\circ}}$C has been reported [18]. The introduction of an intermediate passivation layer with a low melting temperature, such as Sn that has a low melting point, could reduce the temperature required for bonding to 360 ${}^{{}^{\circ}}$C [19]. The intermediate layer acted as a barrier layer to prevent the formation of the native oxide layer. The Sn layer was melted away before the bonding process to release the fresh Al surface for the thermocompression bonding.

Another alternative is by introducing impurity in the bonding layer, such as Cu [20]. The Cu impurity was introduced in the sputtering target, which was used to deposit the bonding frame material. By the introduction of such an impurity and deposition process optimization, including the under layer and deposition temperature adjustment, a bonding surface with less than 2 nm surface roughness was achieved, which enabled the wafer bonding at 300 ${}^{{}^{\circ}}$C. The combination of impurity and noble metal passivation layer has led to a successful bonding even at 250 ${}^{{}^{\circ}}$C bonding temperature [21]. However, the introduction of such an impurity reduces the merit of using Al as a CMOS compatible material. To close the gap, a technique beyond the conventionals is therefore demanded.

To compensate for microstructural variation on wafers with surface microstructures, a thick bonding frame is required. The electroplating process, which can realize several µm thick film with high deposition rate, is more suitable for this purpose than the physical vapor deposition techniques. Using an electroplated bonding frame, surface activated bonding (SAB) technology has been developed to reduce the bonding temperature [22]. In the SAB, the to-be-bonded wafers are activated by Ar plasma consecutively just before the bonding process, which is carried out in the same vacuum chamber. The contaminants on the bonding surface are removed by the Ar plasma and the process enabled the diffusion bonding with a strong adhesion [23,24]. The electroplated frames were polished prior to the bonding sequences to reduce the surface roughness of the bonding interface. However, a specific tool, which enables plasma processes and bonding sequentially at the same chamber is required to perform SAB.

Another class of bonding technique is by applying the fly-cutting to the electrochemically deposited thick films, such as Au and Cu [25,26]. The diffusion bonding was enabled by the smooth surface upon fly-cutting planarization process using a diamond bit, which also refined the surface microstructure. Since the diffusion energy at the grain boundary is lower compared to the bulk grain, the fine microstructure enabled the wafer bonding at a low temperature [26]. However, similar to the previous method, this method requires an additional tool which sometimes is not available.

To compensate the surface roughness of the electrochemically deposited metallic bonding frames, a pressure concentrating structure at the counter wafer can also be utilized [27,28]. A high localized pressure by groove structure at the counter wafer induces a large mechanical deformation, which can also be utilized to intentionally break the native oxide of the electrochemically deposited bonding frame, i.e., press marking.

Al deposition in semiconductor processes has been traditionally performed by either sputtering or evaporation. The electroplating process to deposit Al has been challenging due to its negative standard potential of Al^3+^/Al couple of –1.67 V vs. normal hydrogen electrode. Such a value caused Al to have a high tendency to oxidize under exposure to the water, which is the most common electroplating medium. To perform Al electroplating, the ionic liquid has been introduced as the medium [29]. Past studies have been focused on the development of compatible ionic liquid as a solvent and the deposition parameters [30,31]. The emerging applications of the developed process include the anti-corrosion coating [32,33] and Al-ion battery [34,35]. Recently, the technique has been demonstrated to deposit a thick Al film microstructure [36] and various properties of the deposited film have been characterized [37,38]. In this study, the electroplated Al is implemented as a part of the semiconductor microfabrication process. A press marking technique using the electroplated Al thick film is proposed to answer the demand of low-temperature Al-Al bonding. The press marking technique involves mechanical squeezing of the electroplated Al bonding frame, which helps to release the fresh Al surface for bonding with the counter wafer.

## 2. Materials and Methods

### 2.1. Specimen Preparation

**Figure 1:**
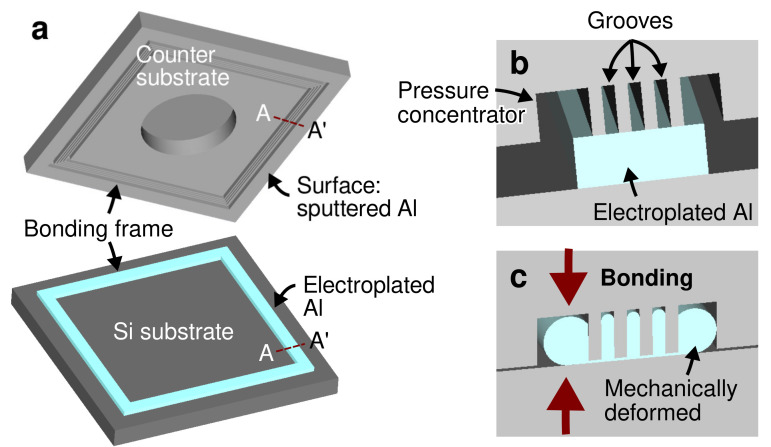
(**a**) Schematics of the test vehicle consisting of a Si substrate with electroplated Al and a counter substrate with grooves as the bonding frames. Enlarged schematics of the bonding interface cross-section A-A’ in-contact (**b**) before and (**c**) after the bonding process.

Al readily forms a native oxide layer on its surface upon exposure to the atmospheric environment. The Al oxide is one of the well-known stable metal oxide forms, which requires a high energy to be broken. In the press marking method, a high localized bonding pressure is used to mechanically break the native oxide layer and other possible contaminants, thus releasing the fresh metallic bonding interfaces. The concept of the bonding process is illustrated in Figure 1. The high local pressure is implemented through the groove structure as the bonding frame at the counter substrate. Such a technique enabled atomic diffusion between the freshly released metallic surfaces, which is the driving mechanism of the bonding process, even at a low temperature elevation [39].

**Figure 2:**
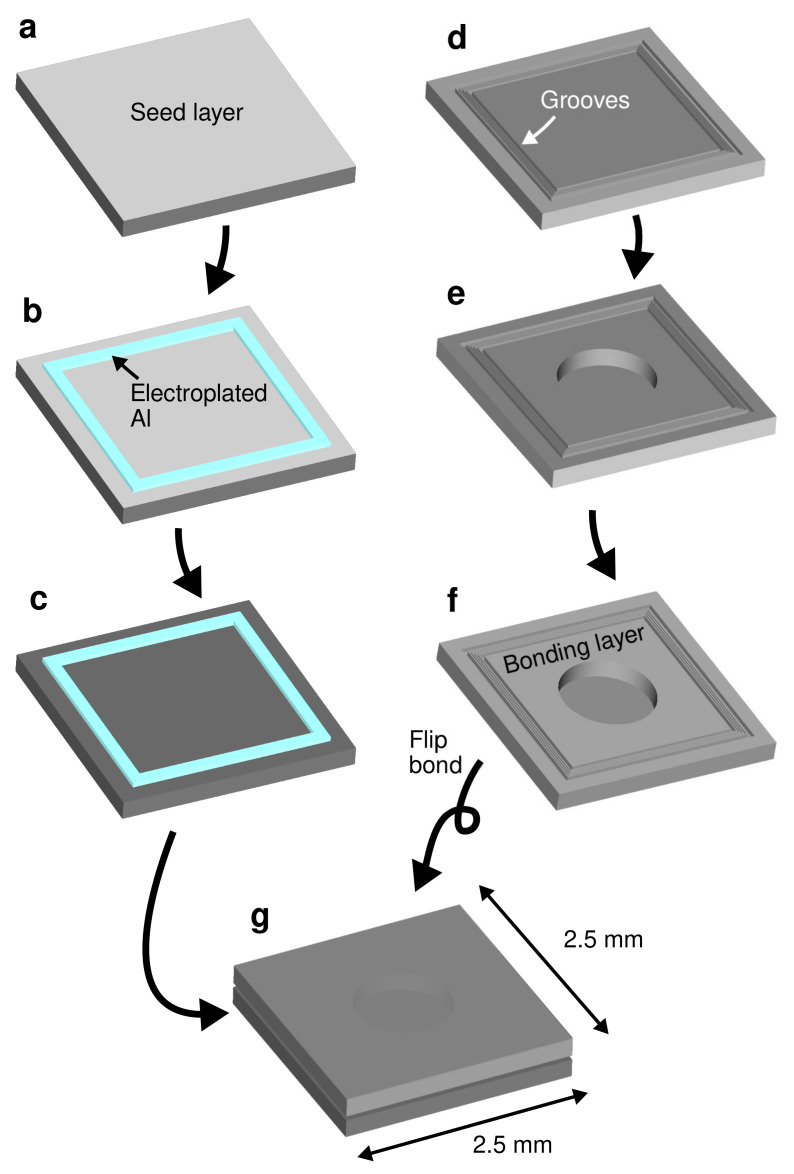
Fabrication process of the test vehicle. (**a**) Electroplating seed layer deposition. (**b**) Al bonding frame electroplating. (**c**) Seed layer removal. (**d**) Groove fabrication on a counter SOI substrate. (**e**) Diaphragm fabrication. (**f**) Bonding layer deposition. (**g**) Wafer bonding.

**Figure 3:**
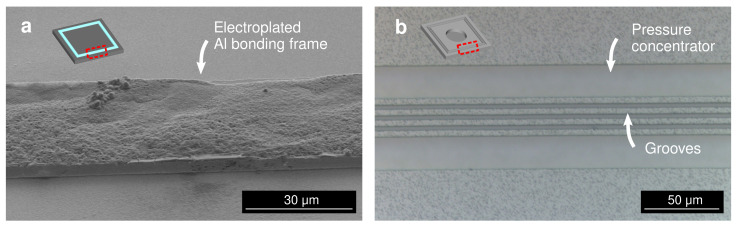
(**a**) Scanning electron micrograph of the electroplated Al bonding frame. (**b**) Optical micrograph of the grooves as the bonding frame at the counter substrate.

Figure 2 illustrates the fabrication process of the test vehicle. A layer of Ti/Pt (20/180 nm) was initially deposited on a Si substrate using a magnetron sputtering, as shown in Figure 2a. Each of the Ti and Pt layers act as an adhesion promotion layer and an electroplating seed layer, respectively. Pt was selected as the seed layer due to its process stability as a noble metal, yet it does not react or diffuse to the electroplated Al film. A positive tone photoresist AZ P4620 (MicroChemicals GmbH, Ulm, Germany) of around 10 µm thickness was patterned on the substrate to define the bonding frames. The Al electroplating process was then performed selectively from a chloroaluminate ionic liquid electrolyte using the patterned photoresist as the mold. A commercially available AlCl_3_-1-ethyl-3-methylimidazolium chloride ([EMIm]Cl) with 3:2 concentration ratio (EP-0001, IoLiTec GmbH, Germany) was used as the electroplating electrolyte with 0.5 g/L 2-chloronicotinyl chloride additive to improve the uniformity of the deposit [37,38]. The electroplating process was performed using a 2-electrode system with a 99.9% Al plate (Nilaco Corp., Tokyo, Japan) as the counter electrode. A direct current of 10 mA/cm^2^ was applied during the deposition in a 36 ${}^{{}^{\circ}}$C bath temperature under a continuous magnetic stirring. The electroplating process was carried out inside a glovebox with a continuous flow of a dry N_2_ gas. Figure 2b illustrates the electroplated Al bonding frame after photoresist stripping. The frame width was either 50 or 30 µm and the thickness was approximately 8 µm. The frame formed a square with 2 mm side length on a single test vehicle chip with a dimension of 2.5 mm × 2.5 mm square. Finally, the seed layer which was not covered by the electroplated film was removed by Ar ion beam milling, as shown in Figure 2c. The fabricated Al bonding frame is displayed in Figure 3a.

A silicon-on-insulator (SOI) substrate was used as the capping substrate. On the handle layer of the SOI substrate, a bonding frame consisting of 3 grooves with the width ranging from 3 to 4 µm, the pitch ranging from 6 to 7 µm and the depth of approximately 5 µm were fabricated by Bosch process Si deep reactive ion etching (DRIE), as shown in Figure 2d. In addition, two wide grooves to enhance the local bonding pressure were fabricated at each sides of the grooves. The illustration of the groove structure is reflected in Figure 1. The total width of the bonding frame on the counter substrate side was designed as 70 µm.

Following the groove formation, a circular diaphragm was patterned by Bosch process Si DRIE on the handle layer of the SOI substrate. The diaphragm holds as a tool to confirm the vacuum sealing of the bonded package. The 400 µm-thick handle layer was etched to the buried oxide (BOX) layer. The 1 µm BOX layer was then etched through using a 49% concentrated hydrofluoric acid (HF) solution. The resulting structure of 10 µm-thick diaphragm is illustrated in Figure 2e. The structures were then covered by Ti/Al (20/1000 nm) layer by the magnetron sputtering as illustrated in Figure 2f. The Ti and Al layers act as the adhesion promotion and the bonding layers, respectively. The groove structures for bonding frame are depicted in Figure 3b. To minimize the influence of unintentional process condition variation, the test vehicle chips were processed in the same substrate.

Finally, both substrates were bonded together as illustrated in Figure 2g. The bonding process was carried out by a local pressure of 250 MPa with different bonding temperatures in a vacuum environment using a commercially available bonding equipment (SB6e, SÜSS MicroTec GmbH, Germany). The local pressure was determined according to the surface area which are initially in contact with each other in between the electroplated Al bonding frame and the groove structure. The bonding force and temperature was applied continuously for 40 min. Each bonded substrate contains 36 test vehicle chips.

### 2.2. Evaluation

**Figure 4:**
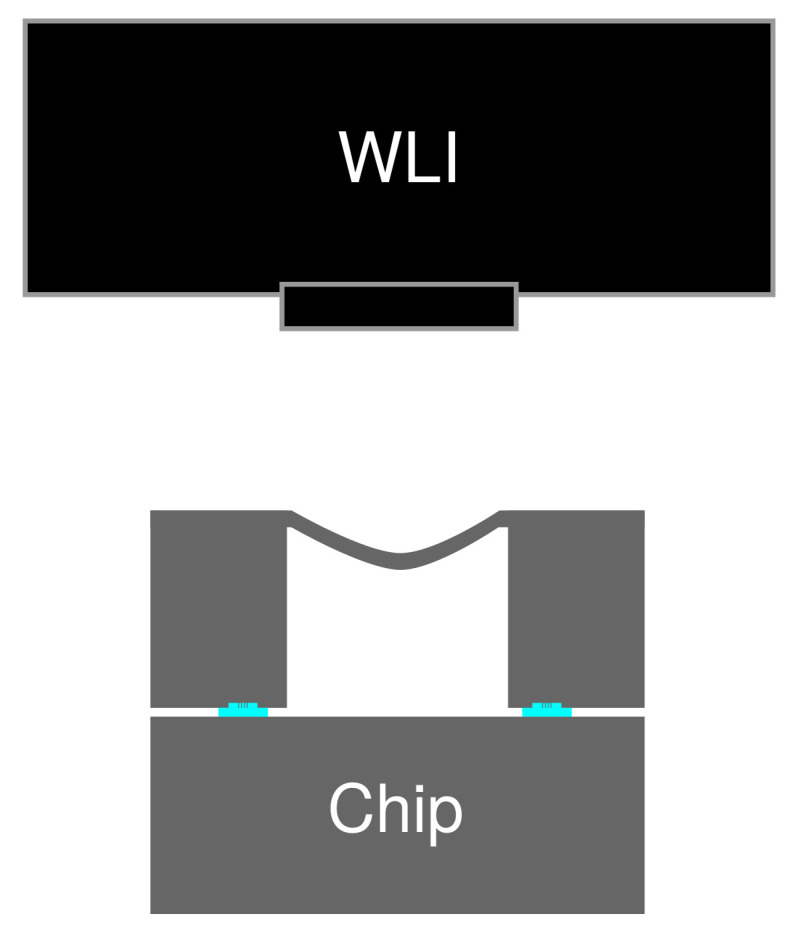
Schematics of the surface topography measurement setup for sealed cavity pressure evaluation.

Along and after the process, the samples were optically observed using an optical microscope (DM4000M, Leica Camera AG, Wetzlar, Germany) and a scanning electron microscope (SEM, SU-70, Hitachi Ltd., Tokyo, Japan). The pressure of the sealed cavity was measured utilizing the 10 µm-thick diaphragm of the wafer bonding test vehicle. The deformation of the diaphragm membrane was measured by a white light interferometry surface topography measurement system (MSA-500, Polytec GmbH, Waldbronn, Germany). The schematics of the evaluation setup is illustrated in Figure 4. The sealed cavity pressure was estimated according to the deflection of circular membrane theory [40]. The deflection of a circular plate, ω, is proportional to the pressure difference between its opposing sides, and can be mathematically represented as,

(1) $$\omega \left(r\right)=\frac{\Delta P}{64D}{\left(a^{2}-r^{2}\right)}^{2},$$

where ΔP is the pressure difference between the opposing surfaces, *a* is the radius of the plate, and *r* is the radial distance from the center of the plate. The material-dependent flexural rigidity, *D*, is given by,

(2) $$D=\frac{Et^{3}}{12(1-v^{2})},$$

where *E* is Young’s modulus of the material of the plate, *t* is the plate thickness, and *v* is the Poisson’s ratio of the material. The atmospheric pressure of $10^{5}$ Pa is set as the reference pressure and the maximum deflection at the center of the diaphragm, i.e., the deflection at r=0, was used for the evaluation.

The leak rate of the package, *L*, was estimated as,

(3) $$L=\frac{P_{\mathrm{cavity}}V_{\mathrm{cavity}}}{\Delta T},$$

where the sealed cavity pressure, $P_{\mathrm{cavity}}$, was estimated as previously explained, the sealed cavity volume, $V_{\mathrm{cavity}}$, is approximately 0.314 mm^3^ and ΔT was the elapsed time between each measurements.

**Figure 5:**
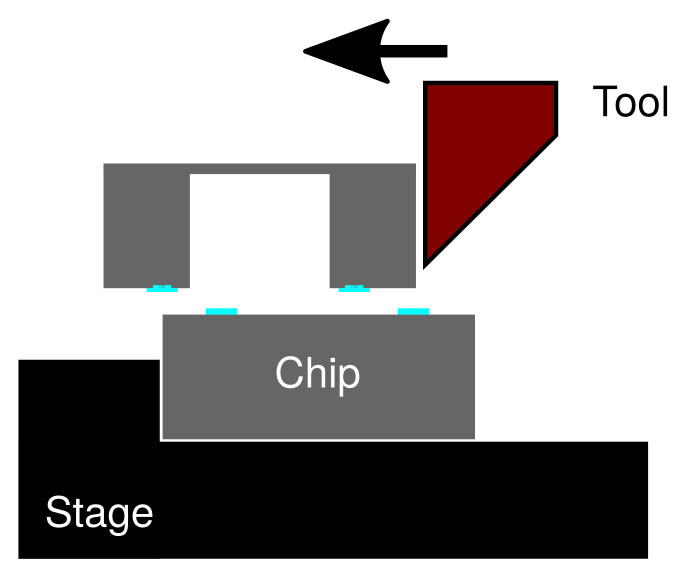
Schematics of the die shear strength testing setup.

The bonded substrates were then diced to single chips. Then, a die shear strength tester (PTR-1101, Rhesca Co., Ltd., Tokyo, Japan) was used to evaluate the bonding strength of the bonded chips. The test was performed by fixing one of the bonded substrates while pushing the other substrate in the horizontal direction. The fracture force was recorded during the experiment. The schematics of the evaluation setup is illustrated in Figure 5. The fracture surface was then observed using a SEM. The shear strength, τ, was determined from the fracture force, $F_{\mathrm{frac}}$, and the projection of the bonding area, *A*, as

(4) $$\tau =\frac{F_{\mathrm{frac}}}{A}.$$

## 3. Results and Discussion

### 3.1. Bonding Mechanism

The bonding process was carried out under temperatures of 450 ${}^{{}^{\circ}}$C or lower with 100 ${}^{{}^{\circ}}$C interval. The 450 ${}^{{}^{\circ}}$C bonding temperature is the typical value to achieve a reliable bonding interface using the standard Al thermocompression bonding technique [16,17]. Using the proposed press marking technique, reliable bonding interfaces were obtained using the bonding temperature down to 250 ${}^{{}^{\circ}}$C. The bonding process at the temperature of 150 ${}^{{}^{\circ}}$C was unsuccessful, which could be attributed to the poor atomic interdiffusion at the bonding interface at such a low temperature.

**Figure 6:**
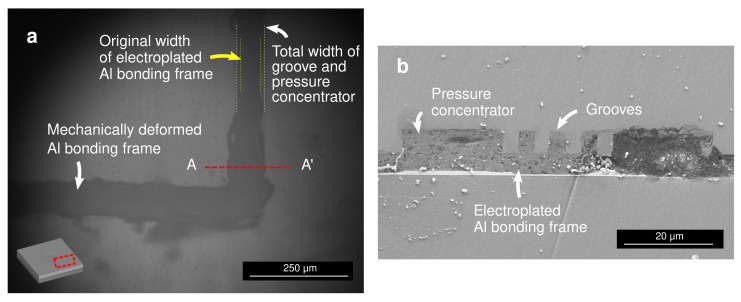
(**a**) Top-view infrared micrograph and (**b**) A-A’ cross-sectional scanning electron micrograph of the bonded substrates.

Figure 6a displays the infrared micrograph taken from above the bonding frame after the bonding process. In the infrared wavelength Si is transparent, which enabled the observation of the mechanically deformed Al bonding frame through the Si substrate. The cross-sectional scanning electron micrograph around the bonding frame is shown in Figure 6b. These observations validated the proposed bonding mechanism, in which the electroplated Al bonding frame was mechanically squeezed to penetrate into the grooves. During the mechanical squeezing process, fresh pure Al surface emerged at the bonding interface, which enabled the atomic interdiffusion at the low temperature.

The bonding mechanism is analyzed by a finite element analysis using a commercially available software Femtet (Murata Software Co., Ltd., Yokohama, Japan). The initial contact between the electroplated Al bonding frame and the grooves at the opposing wafer is simulated using the available 2-dimensional stress analysis solver. The model size is exactly the same as the experimental bonding test vehicle, with the exception of the Si substrate thickness, which was reduced to reduce the computational load. The simulation was carried out using triangular mesh of 0.1 µm size. Convergence was confirmed under these conditions. The mechanical properties of the electroplated Al are in accordance to previous studies, the elastic modulus was 77 GPa and the Poisson’s ratio was 0.34 [37,38]. The material properties of the grooves are those of the single crystalline Si.

As the boundary conditions in the simulation, a normal pressure of 100 MPa was applied to the top of the Al bonding frame and the bottom surface of the Si was fixed. The applied normal pressure in the simulation is equivalent to the applied local force during the experimental bonding process. The top corners of the Al bonding frame was fixed in the horizontal axis direction, which correspond to their positions on the Si substrate. The contact between the Al and Si surfaces was simulated to estimate the bonding mechanism. Through the simulation, a local stress of around 1.5 GPa was observed in the Si grooves, which is far below the hardness of the single crystalline Si. Meanwhile, a local stress of around 1 GPa was observed in the electroplated Al film, which is equivalent to the hardness of the electroplated Al. The bonding occurs by the concentrated local stress which breaks the Al bonding frame and squeeze it in the grooves of the Si.

**Figure 7:**
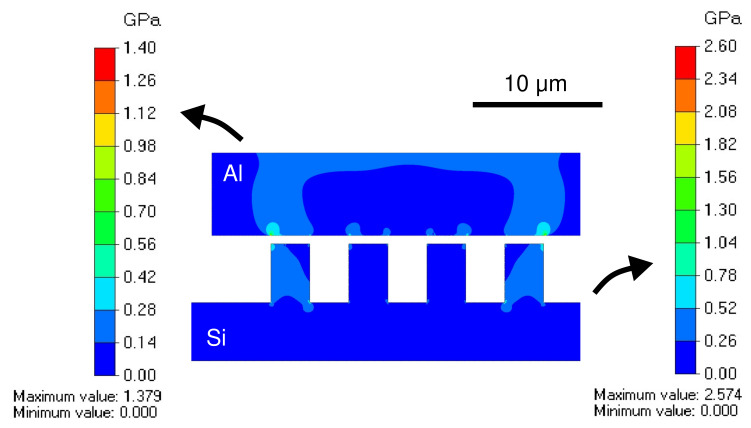
Finite element analysis of the cross-section showing the stress distribution along the bonding frame at the beginning of the bonding process.

The von Mises stress distribution along the bonding frame during the initial step of the bonding is illustrated in Figure 7. The stress propagates through the contact area to both materials with a more widespread influence on the electrochemically deposited Al due to its softness in comparison to the single-crystalline Si at the groove site. Higher concentrated stress was observed at the corners of the grooves, which indicates the origin of the deformation of the bonding frame during the proposed bonding process.

### 3.2. Hermeticity

**Figure 8:**
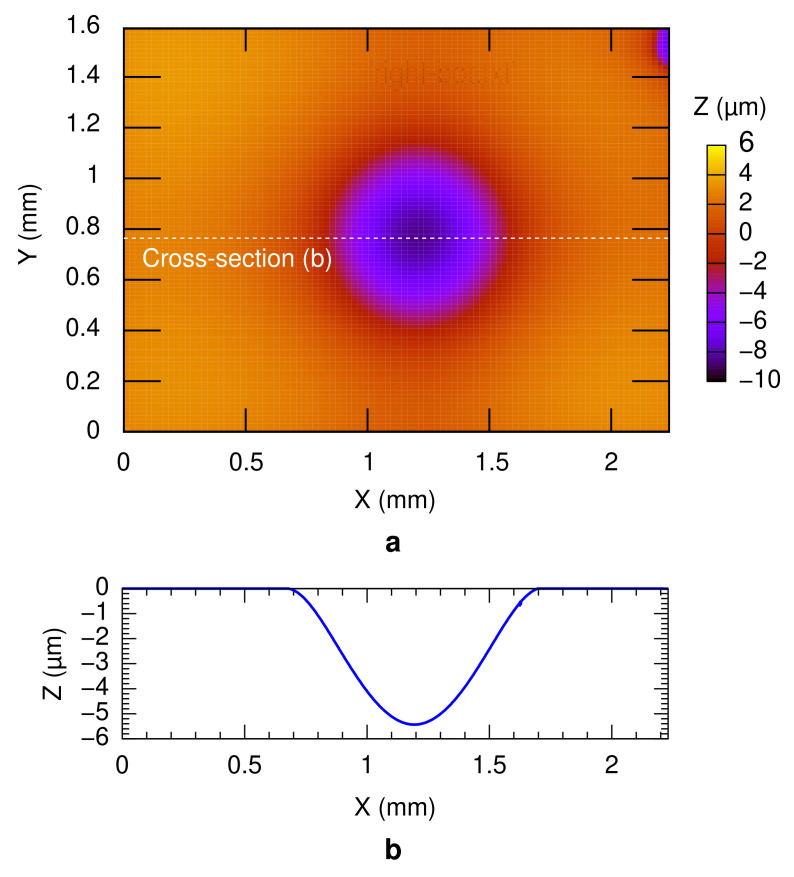
(**a**) Surface profile of the diaphragm after wafer bonding process. (**b**) Cross-sectional 2D surface profile from (**a**).

Hermeticity of a bonded package is an important parameter which determines the long-term quality of a MEMS device. The vacuum sealing of the technology was evaluated by measuring the sealed cavity pressure using the deflection of the diaphragm at the counter substrate in the bonding test vehicle. The typical deflection profile of the diaphragm under the atmospheric pressure is depicted in Figure 8. The measurements were performed 2 h after the bonding experiment. A maximum deflection of around 5 µm was obtained from the cross-section, as shown in Figure 8b. The sealed cavity pressure was estimated accordingly as around 59–88 kPa, for all bonding conditions. Higher vacuum sealing might be possible by introducing a heat pre-treatment prior to the bonding process and/or a thin film getter material inside the bonded packages [25,41]. For a more precise sealed cavity pressure evaluation, the diaphragm can be subjected to the zero-balance method [42]. In the zero-balance method, the deformation of the diaphragm is measured inside a pressure-controllable vacuum chamber at different pressures. The cavity pressure is identical to the chamber pressure when the diaphragm becomes flat. However, in this study, the zero-balance method was not performed due to the high sealed cavity pressure, which voids the necessity of precise sealed cavity pressure measurement. Other cavity pressure measurement methods include the fabrication of a resonator [43,44] or a micro-pirani gauge [45,46,47]. The value of the sealed cavity pressure was then translated to the leak rate of less than 3.8 $\times \;10^{-9}$ Pa m^3^ s^−1^, regardless of the bonding condition.

### 3.3. Dicing Yield

**Figure 9:**
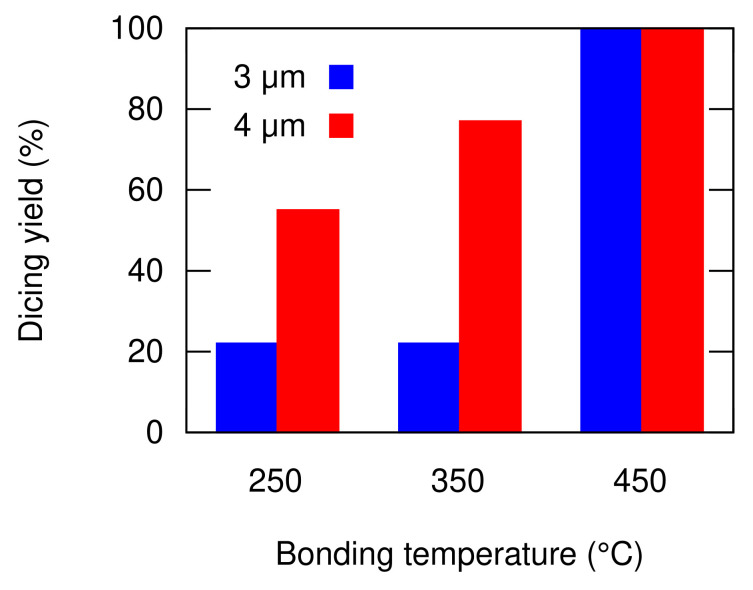
Bonding yield after wafer dicing with respect to the groove size and bonding temperature.

After the bonding process, the bonded substrates were diced to single test vehicle chips. The dicing process often induces shear stress to the bonded package. The stress causes the bonded packages with low quality to peel-off. Therefore, the survival ratio of the bonded packages after the dicing process reflects the bonding quality. The dicing yields of the bonded packages with 12 different conditions are depicted in Figure 9. For each condition, 9 chips were prepared. The dicing yield at 450 ${}^{{}^{\circ}}$C bonding temperature was 100% for all the design parameters. This indicates that the diffusion between the bonding surfaces occurred sufficiently to realize a high reliability bonding interface. The temperature is also the typical bonding temperature for Al thermocompression bonding [16,17]. The overall dicing yield decreases with the bonding temperature. This indicates that the bonding quality deteriorates with the decrease in the bonding temperature.

The dicing yield gradually decreased with the bonding temperature. The one with a wider groove width (4 µm) gave a higher yield with 77% and 55% at the bonding temperature of 350 ${}^{{}^{\circ}}$C and 250 ${}^{{}^{\circ}}$C, respectively, in comparison to 22% of the narrower groove width (3 µm). This could be an indication that the wider groove provides a better groove penetration area. No successful bonding was obtained at the bonding temperature of 150 ${}^{{}^{\circ}}$C or lower.

### 3.4. Shear Strength

**Figure 10:**
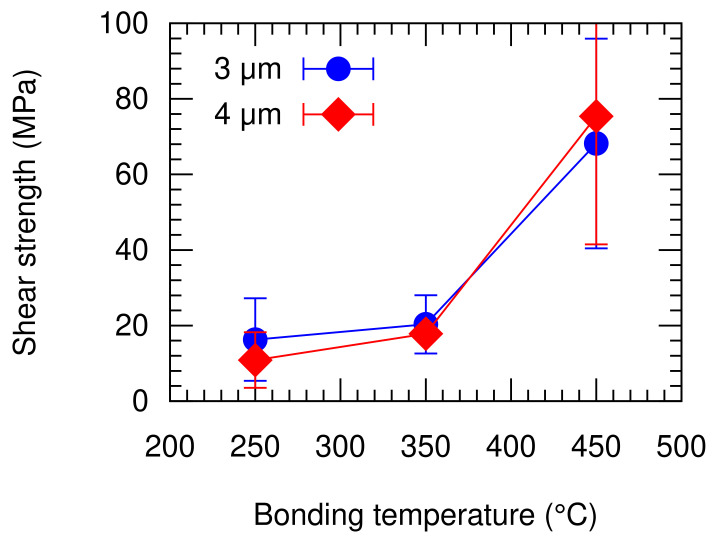
Die shear strength of the bonded packages with respect to the groove size and bonding temperature.

Figure 10 shows the measured die shear strength of the bonded packages with respect to the groove size and the bonding temperature. Around 2–5 chips were tested for each condition, which depends on the number of survived packages from the previous steps. The shear strength of the bonded packages generally decreases with the bonding temperature, irrespective to the groove design. Such a trend is partly in agreement with the dicing yield result mentioned in the previous section. This is also an indication that the bonding quality deteriorates with the decrease in the bonding temperature.

At 450 ${}^{{}^{\circ}}$C bonding temperature, the mean shear strengths were 68 MPa and 75 MPa for the specimens with 3 µm and 4 µm groove width, respectively. All the packages bonded at 450 ${}^{{}^{\circ}}$C showed a shear strength of over 40 MPa. At the lower bonding temperature of 350 ${}^{{}^{\circ}}$C, the mean shear strengths deteriorated to 20 MPa and 18 MPa, with 3 µm and 4 µm groove width, respectively. At the lower bonding temperature of 250 ${}^{{}^{\circ}}$C, the figures further dropped to 16 MPa and 11 MPa for the devices with 3 µm and 4 µm groove width, respectively. The groove width difference did not significantly influence the bonding shear strength. On the other hand, the bonding strength of Al–Al diffusion bonding has been reported to deteriorate with the decreasing bonding temperature [18], which is in agreement with this study. The bonding strength has been associated with the quality of the atomic diffusion at the bonding interface [18]. In general, the die shear strength of the bonded packages is comparable to those of the established technologies where Al was employed as listed in Table 2. The fracture mode of the bonded packages is analyzed more in depth in the following section.

### 3.5. Fracture Surface

The fracture surface of the shear tested packages were observed by a SEM. The fracture surface typically indicates the weakest part on the package which is damaged by the shear test in comparison to the other parts. The fracture surface or the delamination path is identified by the SEM observation of the bonding frame of the shear tested packages. Its observation often provides knowledge on the fracture mechanism. Such information is useful to further improve the reliability of the bonding technology for practical applications.

**Figure 11:**
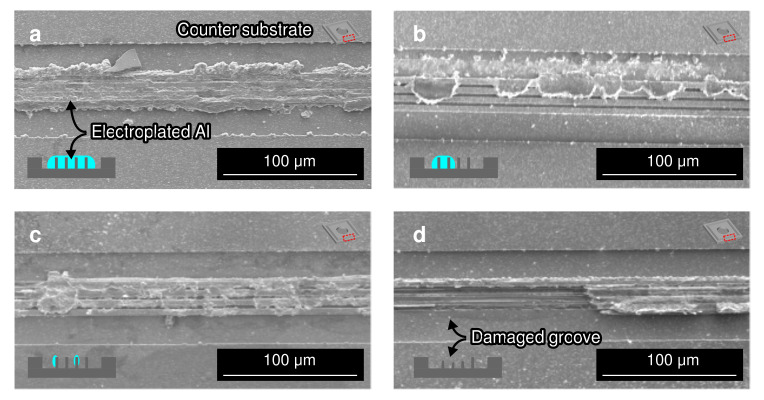
Scanning electron micrographs of the fracture surface after shear test of the bonded packages at (**a**) 450 ${}^{{}^{\circ}}$C, (**b**) 350 ${}^{{}^{\circ}}$C, and (**c**,**d**) 250 ${}^{{}^{\circ}}$C bonding temperatures.

Figure 11a shows the typical fracture surface of the packages bonded at 450 ${}^{{}^{\circ}}$C bonding temperature. At such a high bonding temperature, a relatively high bonding strength for all conditions regardless of the groove design. The typical fracture surface was at the bulk electroplated Al. These results confirm that the weakest part of the bonded package was not the bonding interface, which suggests an adequate atomic interdiffusion between the electroplated Al and sputter-deposited Al on the counter substrate at the bonding interface. In addition, the fracture surface also indicates that the electroplated Al bonding frame was squeezed and penetrated inside the grooves. The amount of electroplated Al penetrated into the Si groove also contributed to enhance the bonding strength. The lateral spread of the Al bonding frame was 30–40%. According to the observation, the bonding frames were squeezed from 30 µm to around 40 µm.

Figure 11b shows the dominant fracture surface of the packages bonded at 350 ${}^{{}^{\circ}}$C bonding temperature. In this condition, a lower die shear strength and dicing yield were obtained in comparison with those bonded at 450 ${}^{{}^{\circ}}$C. The fracture surface indicate that the fracture occurred partially at the bulk electroplated Al and at the bonding interface. Such is an indication that in some areas, the bonding interface is stronger than the bulk electroplated Al, and in the other areas the bonding interface is weaker than the bulk electroplated Al. The lateral spread of the Al bonding frame was similar to the packages bonded at 450 ${}^{{}^{\circ}}$C bonding temperature at 30–40%. The bonding frames were also squeezed from 30 µm to around 40 µm.

The dominant fracture surface of the packages bonded at 250 ${}^{{}^{\circ}}$C bonding temperature is shown in Figure 11c. At such a bonding temperature, a lower die shear strength and dicing yield were obtained in comparison with the packages bonded at 450 ${}^{{}^{\circ}}$C and 350 ${}^{{}^{\circ}}$C. The fracture surface was dominant at the bonding interface. This shows that at lower bonding temperature, the weakest point shifts from the the bulk electroplated Al to the bonding interface. Some peeled substrates also showed a fractured interface at the groove, as shown in Figure 11d. Such a fracture mode was not observed at the substrates bonded at 450 ${}^{{}^{\circ}}$C and 350 ${}^{{}^{\circ}}$C bonding temperatures. The grooves could be fractured either during the bonding process or the die shear testing, or as a combination of both. The initial fracture on the groove during the bonding process propagates during the die shear strength testing and became the weakest point in some parts. The fracture during the bonding process can also be attributed to the high pressure applied to the structure against the electroplated Al bonding frame at the low temperature. The low contribution of the bonding temperature in softening the Al film led to the fracture at the groove structure. Due to this effect, the electroplated Al frame might also penetrated and fill the grooves only partially. The existence of gap in the groove also has a potential to reduce the bonding strength.

The lateral spread of the Al bonding frame was similar to the packages bonded at 450 ${}^{{}^{\circ}}$C and 350 ${}^{{}^{\circ}}$C bonding temperatures at 30–40%. The bonding frames were also squeezed from 30 µm to around 40 µm. This also indicates that the press marking technique was able to mechanically deform the electroplated Al frame at the same amount with 250–450 ${}^{{}^{\circ}}$C bonding temperatures. However, the penetration of the electroplated Al into the groove, which is the driving mechanism of this bonding technique, deteriorated with the decrease in the bonding temperature. Such is also an indication that the combination of the squeezed Al penetration into the groove and the atomic diffusion was insufficient to perform a wafer bonding at temperatures of 150 ${}^{{}^{\circ}}$C or lower.

## 4. Conclusions

In this study, a wafer-bonding technology using electroplated Al film has been demonstrated. The mechanism is based on the press marking, in which the electroplated Al bonding frame was mechanically deformed by the localized high pressure transmitted through the groove structure at the counter substrate. Such a method has enabled the bonding at a temperature as low as 250 ${}^{{}^{\circ}}$C without requiring any additional passivation layer or impurity. The bonding quality corresponds to the bonding temperature. The bonding die shear strength was around 8–100 MPa depending on the bonding temperature, which is comparable to the existing technologies. The leak rate to the bonded packages were in the order of $10^{-9}$ Pa m^3^ s^−1^. The proposed technique holds a great potential to pave the way for Al wafer bonding at low temperature as a CMOS-friendly process for 3D heterogeneous integration and packaging. Potential applications include the CMOS-MEMS integrated tactile sensor [48], encapsulation of inertial sensors [49] and heterogeneous integration of multiple substrates [50].

## Figures and Tables

**Table 1:** Existing metal-based solid-state wafer bonding technologies.

Material	Deposition	Features
Au	Sputtering/evaporation	+ Smooth bonding surface + Stable against process conditions – Expensive material – Requiring diffusion barrier layer
	Electroplating	+ Compensating structural variation on the device – Requiring surface planarization step
Cu	Sputtering/evaporation	+ Smooth bonding surface + Low cost material – Requiring either pre-treatment prior to bonding or high bonding temperature – Requiring diffusion barrier layer
	Electroplating	+ Compensating structural variation on the device – Requiring surface planarization step
Al	Sputtering/evaporation	+ Smooth bonding surface + Low cost material – Requiring high bonding temperature

**Table 2:** Comparison with the state-of-the-art Al wafer bonding technologies.

Technology	Temp. (${}^{{}^{\circ}}$C)	Strength (MPa)	Note	Ref.
Ultra thin film	RT	NA	Consecutive deposition-bonding	[11]
Sputtered film	450	339	Sputtered thin film	[17]
	300–450	11–80		[18]
Surface passivated	360–390	32–209	Sn passivation	[19]
Impurity included	300–500	NA	Cu impurity	[20]
Impurity + passivation	250	30	Cu impurity, Pd passivation	[21]
This study	250–450	8–100	Pure Al press marking	

## Data Availability

All data are available in the manuscript.
